# Fear of missing out (FOMO) as a predictor of smartphone addiction and mental health outcomes among University Students in Dhaka, Bangladesh: A cross-sectional study

**DOI:** 10.1371/journal.pone.0352129

**Published:** 2026-08-12

**Authors:** Ryadul Alam, Ifteker Uddin Sakib, Juned Hussain Chowdhury, Sadia Akter, Md Mushfiqur Rahman Porosh, Md Tajuddin Sikder

**Affiliations:** Department of Public Health and Informatics, Jahangirnagar University, Savar, Dhaka, Bangladesh; PLOS: Public Library of Science, UNITED KINGDOM OF GREAT BRITAIN AND NORTHERN IRELAND

## Abstract

Growing dependence on smartphones has created new psychological and behavioral challenges. This issue is particularly pressing among young adults who are at a critical stage of emotional and academic development. This study aims to explore the relationship between fear of missing out, mobile phone addiction, and mental health status among university students in Dhaka. Participants were recruited from both public and private universities through a multistage stratified random sampling approach to conduct a cross-sectional study. A total of 500 undergraduate students aged 18–25 years were included. Data was collected using a structured, self-administered online questionnaire comprising validated Bangla versions of the FoMO Scale, Smartphone Addiction Scale, and the Depression, Anxiety, and Stress Scale (DASS-21). More than half of the participants (56.8%) experienced a moderate level of Fear of Missing Out (FoMO), while 8.8% reported a high level of FoMO. About one-fifth (21.4%) met the criteria for smartphone addiction. Psychological distress was widespread; nearly 70% of respondents reported depressive symptoms, over half (54.6%) experienced extremely severe anxiety, and more than 60% had moderate to severe stress. Participants with depression (OR = 7.01, 95% CI: 4.27–11.47), anxiety (OR = 5.61, 95% CI: 3.36–9.35), and stress (OR = 9.69, 95% CI: 6.33–14.84) were significantly more likely to exhibit high FoMO. Smartphone-addicted individuals were 4.46 times more likely to have high FoMO (95% CI: 2.45–8.11, p < 0.001). Additionally, FoMO was significantly linked to late-night smartphone use (p < 0.001) and poor sleep habits (p = 0.046). Addressing FoMO through awareness, digital well-being education, and mental health support within university settings could help reduce smartphone dependency and improve students’ psychological resilience.

## Introduction

Mobile phones are now a part of life in the digitally connected world, and this is particularly true among university students. Due to the popularity of social media websites and the use of smartphones, people have always been kept up to date about the occurrences or happenings in others’ lives. Although this constant connectivity has many positive effects, it has also been associated with another psychological phenomenon: the Fear of Missing Out (FoMO). FoMO is a fear of people living rewarding lives without the individual being present, and this desire to constantly remain connected makes a strong appeal [1]. University students are particularly vulnerable to FOMO due to their high social engagement and developmental stage [2]. Research indicates that high FOMO is associated with increased anxiety and depression, as individuals constantly compare themselves to others and perceive themselves as missing out on social events or experiences [3]. Over the past couple of years, mobile phones have gained widespread use around the world. Although smartphones have the benefits of social networking [4] and contribute to productivity, an emerging literature notes that a significant portion of individuals excessively use the devices in ways that disrupt normal operations [5].

Countries like Bangladesh are going through a very fast and mostly unregulated digital transition. Students are becoming more dependent on their smartphones in both academic and social life, and there is little infrastructure to counter the adverse outcomes of overuse [6]. The need to stay socially connected and stay digitally active among university students can be very forceful to the point that it causes an addiction to the use of the smartphone, which can turn out to be a behavioural tendency referred to as mobile phone addiction (MPA). Their high use of smartphones in education, communication, and entertainment is combined with a critical phase of psychosocial development, which is identity formation and academic performance. FoMO-related excessive device use may interfere with this balance, resulting in time mismanagement, sleeping problems, and decreased academic performance [7]. In urban contexts such as Dhaka, widespread smartphone availability, peer influence, and academic pressure further exacerbate this issue [8]. Moreover, problematic smartphone use (PSU) is often associated with mental health challenges, especially depression and anxiety [9]. Studies demonstrate that students with high levels of FOMO and smartphone addiction exhibit higher rates of depressive symptoms, anxiety, and stress [3,10]. The constant engagement with social media and smartphones contributes to sleep disturbances and emotional strain, leading to deteriorating mental health [11]. It is possible that psychological and physiological mechanisms, including excessive reassurance seeking, impulsiveness, and extraversion, can play a role in problematic smartphone use [12]. In addition, psychological and emotional disturbances can negatively impact health outcomes in adolescents and young adults because they are linked to cognitive and physiological changes, including heart rate variability (HRV), a key indicator of adaptive capacity [13]. The interaction between FoMO, mobile phone addiction, and mental health is thus becoming a major concern.

Students in universities are especially susceptible to such influences, as life at that juncture is in transition. FoMO and PSU have always been associated with suboptimal well-being, poor concentration, and lowered quality of life [1,7]. The long-term habits and coping mechanisms are also established at this development level, which is why the habits that the students develop at this stage of smartphone use can have some long-lasting effects on their psychological health. The overuse of digital interactions can further decrease the chances of face-to-face interactions, which are essential in establishing a social support system and emotional stability. Although fear of missing out (FoMO) is consistently linked with problematic device use and poorer mental health in many high-quality studies, the evidence is still uneven in ways that make a focused study among university students necessary. FoMO reliably predicts mobile phone addiction across samples and theoretical models, yet most large syntheses and empirical studies have been conducted in Europe, East Asia, and North America rather than South Asia, limiting cultural generalizability [14]. In Bangladesh, numerous studies show a large proportion of problematic smartphone use and its relevance to sleep issues in a wide range of students. Nonetheless, the majority of studies have mainly concentrated on the use of social media, but not on the general trends of mobile phone dependency [15]. Moreover, digital addiction can only be developed using validated Bangla instruments. Therefore, future studies are required to incorporate rigorously adapted FoMO scales, device-based measures of addiction, and conventional mental health tools. Such studies ought to employ representative samples of universities and should control for the major confounders, including hours of smartphone use, living situation, and socioeconomic status. This may help in creating contextually relevant evidence to guide campus mental health initiatives and digital well-being interventions in Dhaka. This research aims to address the relationship between FoMO, mobile phone addiction, and mental health status among students in a university in Dhaka. Through these relationships, the study will help in the enhancement of the understanding of the behavioural and psychological issues students have to contend with in the digital age and offer suggestions for the prevention of such issues. Besides, it is hoped that this exploration can illuminate the way the stress of always being connected can affect personal health as well as the academic culture at large. Overall, a better understanding of these factors would aid in the creation of interventions, awareness campaigns, and support services that would lead students to more healthy use of technology and safeguard mental well-being and academic performance.

## Materials & Methods

### Study design

**Fig 1:**
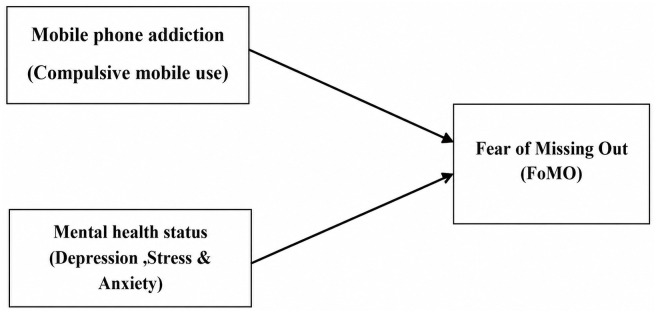
Conceptual framework of the study illustrating the hypothesized influence of mobile phone addiction and mental health status on FoMO.

This study adopted a quantitative cross-sectional design to assess the relationship between Fear of Missing Out (FoMO), mobile phone addiction, and mental health outcomes (depression and anxiety) among university students. The cross-sectional approach allows for the measurement of all variables at a single point in time, making it suitable for exploring associations and identifying prevalence rates within the study population. This study was based on the theoretical assumption that Fear of Missing Out (FoMO) plays a central role in influencing two main outcomes: Mobile Phone Addiction and Mental Health Status (specifically: Depression, Stress, and Anxiety) (Fig 1).

### Study area and population

The study was conducted in Dhaka, the capital city of Bangladesh. Several public and private universities located within Dhaka were selected as study sites, considering the high concentration of undergraduate students who actively use mobile phones and digital platforms. The target population consisted of undergraduate university students (aged ≥18 years), who were currently enrolled in a university in Dhaka city (both public and private universities). These students represent a digitally active demographic that is most likely to experience FoMO and engage in extensive mobile phone use. Participants must be full-time students and capable of providing informed consent.

### Study period

The study was conducted over a period of six months, beginning from July 2025 to December 2025. This period includes time for proposal approval, data collection, data analysis, and report writing.

### Sample size and technique

This study used a multistage stratified random sampling technique to ensure that the sample is representative and free from major biases. First, several public and private universities in Dhaka were randomly selected to ensure institutional diversity. Then, within each selected university, departments were grouped by disciplines such as Health Sciences, Engineering, and Social Sciences, and randomly chosen. From each chosen department, students were further stratified by academic year (first to fourth year), and participants were randomly selected from each year to ensure a balanced sample. This method ensured that students from different types of universities, academic fields, and levels of study are fairly represented. The final sample size, after adjusting for non-response, was 500 at a 95% confidence interval, with a 20% inflation of sample size and a 5% margin of error.

Although a multistage stratified random sampling approach was used to enhance representativeness, potential selection bias may still exist due to non-response and logistical constraints in participant recruitment.

### Data Collection tools & techniques

Data was collected using a self-administered online questionnaire. The survey was hosted on a secure platform and sent to students through their university email to avoid bias from social media sharing. Each student received a unique link that can be used only once to maintain data accuracy and avoid duplicate responses. To make the questionnaire understandable and reliable, previously validated Bangla versions of the Fear of Missing Out (FoMO) Scale, Smartphone Addiction Scale, and DASS-21 (Depression, Anxiety, Stress Scale) were used. These tools were pilot tested on a small group of students before the actual survey to ensure clarity and appropriateness. Participation was completely voluntary and anonymous. No names or identifiable information were collected. The survey remained open for three weeks, and students received up to two gentle reminders to encourage participation. For students who did not have regular internet access, the survey was also made available at university computer labs on specific days. Research assistants were present only to provide technical help, not to influence responses. All collected data were kept confidential, securely stored, and checked for accuracy. This approach was designed to reduce bias and ensure honest responses from participants.

### Data management and analysis plan

Data was processed and analyzed by using computer-based software SPSS version-25 and R programming. Different statistical methods were applied for data analysis. P-value was considered statistically significant when it was less than 0.05 with 95% confidence interval, and chi-square test was done to assess the relationship between outcomes and independent variables.

Binary logistic regression analyses were conducted to identify predictors of study outcomes. Variables associated with the outcome at a significance level of p < 0.20 in bivariate analysis, along with variables identified from prior literature as potential confounders (including age, sex, academic year, socioeconomic status, and relevant behavioral factors), were entered into multivariable logistic regression models.

Adjusted odds ratios (AORs) with 95% confidence intervals (CIs) were reported. Model assumptions were evaluated by checking model goodness-of-fit was assessed using the Hosmer–Lemeshow test.

Any analysis with a p-value of less than 0.05 was statistically significant.

### Ethical approval

The study was conducted in compliance with the guidelines of the Helsinki Declaration of 1975. The study protocol was evaluated and approved by the Biosafety, Biosecurity & Ethical Committee, Faculty of Biological Sciences (Ref No: BBEC, JU/M 2025/08(303)). Participants were fully informed about the study’s purpose, data confidentiality, further utilization of the collected data, and their right to withdraw from the study at any time. Written informed consent was obtained from all participants or their legal authorized representatives.

## Result

### Sociodemographic Characteristics of the participants

A sample of 500 university students was selected, which is representative of a population of young adults, with a minimum age of 23–27 years (79.6%), then 18–22 years (18.6%), and only a minor fraction (1.8%) above 27 years. There was an almost equal gender proportion; 54.2% male and 45.8% female. Regarding marital status, most of the respondents were single (69.2%), 26.4% were married, with a minor percentage (4.4%) divorced or separated. Most of the students studied in private universities (66.2%), whereas 33.8% studied in public. As far as academic fields are concerned, the greatest percentage consisted of the students of sciences and biological sciences (41.6%), business studies (32.0%), and social sciences (25.4%). Regarding the housing, about 37.8% of the respondents resided with their families, 31.4% in rented houses, and 30.8% in hostels. The socioeconomic analysis showed that 35.4% of the participants receive dedication of 15,000–30,000 BDT per month as family income, 29% earn 50,001–70,000 BDT, and 19.6% earn above 70, 000 BDT. Most of them were, therefore, middle-income families, which implies moderate and adequate digital devices and internet access. The physical activity levels of the participants were relatively low. Only 7.6% had to exercise regularly, 61.4% did it occasionally, and 31% never did it. Patterns of sleep durations showed that 40.2 percent of respondents slept below six hours daily, and 59.8 percent slept between six and nine hours, which indicates that insomnia is prevalent among college students- possibly due to nighttime smartphone use. Physical pain was also prominent among respondents, with the most commonly reported symptoms being eye pain (45.6%), neck pain (31.2), followed by shoulder pain (17.6), and hand pain (5.6). BMI distribution showed that 53.2% of the students were within the ideal range, 30.4% overweight, 10.8% underweight, and 5.6% obese (Table 1).

**Table 1:** Demographics information of the participants.

Characteristics	Frequency (n)	Percentage (%)
**Age**		
18–22 years old	93	18.6
23–27 years old	398	79.6
More than 27 years old	9	1.8
**Gender**		
Male	271	54.2
Female	229	45.8
**Marital Status**		
Single	346	69.2
Married	132	26.4
Divorced	20	4.0
Separated	2	0.4
**University Type**		
Public	169	33.8
Private	331	66.2
**Department/ Faculty of Study**		
Science or Biological Science	208	41.6
Business	160	32.0
Social Science	132	25.4
**Current Living Arrangement**		
University Hostel	154	30.8
Rented Home	157	31.4
With Family	189	37.8
**Monthly Family Income**		
15,000–30,000 BDT	177	35.4
30,001–50.000 BDT	80	16
50,001–70,000 BDT	145	29
>70,000 BDT	98	19.6
**Physical Exercise**		
Never	155	31.0
Sometimes	307	61.4
Regularly	38	7.6
**Average Sleeping Hours**		
< 6 hours	201	40.2
6–9 hours	299	59.8
**Experienced Pain**		
Shoulder	89	17.6
Eye	238	45.6
Neck	156	31.2
Hands	29	5.6
**Body Mass Index (BMI)**		
Underweight	54	10.8
Optimal	266	53.2
Overweight	152	30.4
Obese	28	5.6

### Smartphone utilization behaviours of participants

**Fig 2:**
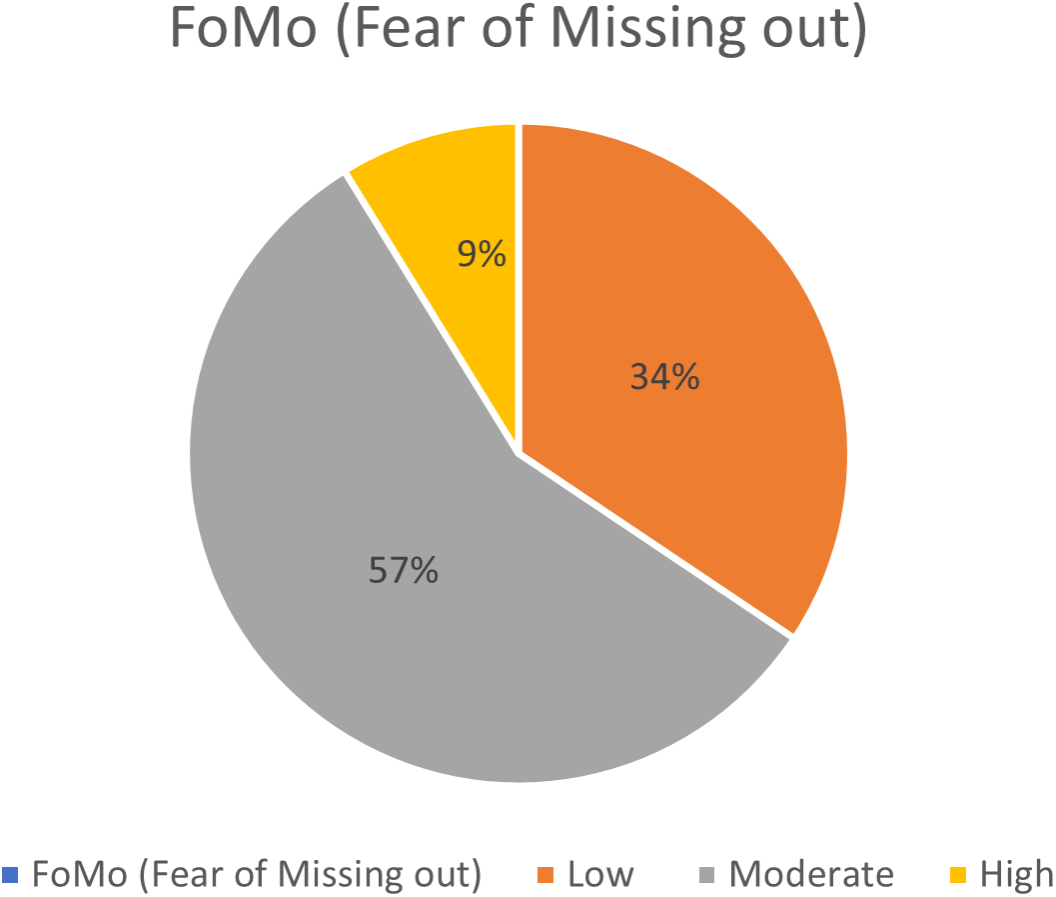
Percentage of FoMo (Fear of Missing Out) among university students.

The data indicate that a substantial proportion of respondents (72.4%) reported that smartphone use keeps them awake at night, whereas only 27.6% stated that their smartphone use does not interfere with their sleep routine. Regarding sleeping habits, 60.0% of respondents reported staying awake after 12 AM, while 40.0% reported going to bed before or up to midnight. Concerning behavioral patterns related to smartphone use, 66.8% of participants admitted using their phones while walking, whereas 33.2% reported avoiding such behaviour. Moreover, 62.6% of the respondents reported using their smartphones while driving, cycling, or riding. A striking 76.1% of participants mentioned using their smartphones during mealtime, suggesting a high level of dependency and integration of smartphone use into daily routines. Regarding the psychological aspect, the analysis of Fear of Missing Out (FoMO) revealed that 56.8% of participants had a moderate level of FoMO, 34.4% exhibited a low level, and only 8.8% demonstrated a high level of FoMO (Fig 2). In terms of smartphone addiction status, 21.4% of respondents were identified as addicted to smartphone use, while the remaining 78.6% were categorized as not addicted (Table 2).

**Table 2:** Distribution of smartphone use behaviors, FoMO, and addiction status among study participants.

Characteristics	Frequency (n)	Percentage (%)
**Is smartphone keep you awake?**		
Yes	362	72.4
NO	138	27.6
**Awaking time**		
Up to 12 AM	200	40.0
After 12 AM	300	60.0
**Using smartphone while walking**		
Yes	334	66.8
No	166	33.2
**Using smartphones while driving, cycling or riding**		
Yes	313	62.6
No	187	37.4
**Using smartphone while eating**		
Yes	381	76.1
No	119	23.8
**FoMo (Fear of Missing out)**		
Low	172	34.4
Moderate	284	56.8
High	44	8.8
**Smartphone Addiction**		
Addicted	107	21.4
Not addicted	393	78.6

### Distribution of Depression, Anxiety & Stress

**Fig 3:**
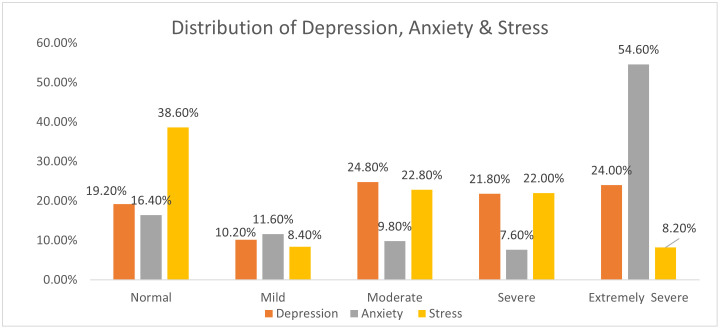
Percentage of depression, anxiety and stress.

For depression, 19.2% of respondents were classified as having normal levels, while 10.2% reported mild, 24.8% moderate, 21.8% severe, and 24.0% extremely severe depression. Regarding anxiety, 16.4% of participants were in the normal range, while 11.6% had mild, 9.8% moderate, 7.6% severe, and 54.6% extremely severe anxiety levels. The high percentage of participants exhibiting extremely severe anxiety (more than half of the total respondents) is alarming and indicates widespread psychological tension and apprehension among the sample. In the case of stress, 38.6% of respondents reported normal stress levels, followed by 8.4% with mild, 22.8% with moderate, 22.0% with severe, and 8.2% with extremely severe stress. Although a relatively higher percentage of participants fell within the normal range compared to depression and anxiety, more than 60% of respondents still experienced varying degrees of stress. Overall, the findings demonstrate that a substantial proportion of participants suffered from elevated levels of depression, anxiety, and stress (Fig 3 and Table 3).

**Table 3:** Distribution of depression, anxiety, and stress among participants.

Categories	Normal n (%)	Mild n (%)	Moderate n (%)	Severe n (%)	Extremely Severe n (%)
**Depression**	96 (19.2)	51 (10.2)	124 (24.8)	109 (21.8)	120 (24.0)
**Anxiety**	82 (16.4)	58 (11.6)	49 (9.8)	38 (7.6)	273 (54.6)
**Stress**	193 (38.6)	42 (8.4)	114 (22.8)	110 (22.0)	41 (8.2)

### Association between FoMo and demographic characteristics

The majority of participants with high FoMO belonged to the 23–27 years age group (81.4%), followed by those aged 18–22 years (16.8%) and above 27 years (1.8%). However, the association between FoMO level and age group was not statistically significant (p = 0.348). Similarly, gender was not found to be a significant factor influencing FoMO level (p = 0.275). In contrast, marital status showed a strong and statistically significant association with FoMO (χ² = 22.364, p < 0.001). The majority of respondents with high FoMO were single (75.6%), compared to 20.4% who were married and 4% who were divorced. Single participants were more than twice as likely to report high FoMO than married respondents (OR = 0.41, 95% CI: 0.26–0.61, p < 0.001). The type of university (public vs. private) did not exhibit a significant association with FoMO level (p = 0.821), indicating that both groups were similarly affected. However, academic discipline was significantly associated with FoMO (χ² = 27.906, p < 0.001). Students from science and biological science faculties demonstrated a much higher proportion of high FoMO (50%) compared to those from social sciences (23.2%) and business faculties (26.8%). The odds of experiencing high FoMO were 2.74 times greater among students from science-related departments compared to those from social science backgrounds (95% CI: 1.70–4.43, p < 0.001). Living arrangement was not significantly associated with FoMO level (p = 0.080), though students living in university hostels (34.1%) and rented homes (29.6%) reported relatively higher levels of high FoMO compared to those living with family (36.3%). A highly significant association was found between monthly family income and FoMO level (χ² = 33.827, p < 0.001). Respondents from families with an income between 15,000 and 30,000 BDT were more likely to exhibit high FoMO (42.7%) compared to those from higher-income families. The odds of having high FoMO were 4.64 times higher among students from low-income families (95% CI: 2.71–7.95, p < 0.001). Regarding lifestyle factors, neither exercise frequency (p = 0.447) nor sleep duration (p = 0.583) showed a statistically significant relationship with FoMO level. Similarly, body mass index (BMI) categories were not significantly associated with FoMO (p = 0.818) (Table 4).

**Table 4:** Association between FoMo with socio demographics characteristics.

Variable	Low FoMo	High FoMo	Χ^2^	P value	OR (95% CI)	P value
**Age (years)**						
18–22	38 (22.1%)	55 (16.8%)	2.114	0.348	Reference	
23–27	131 (76.2%)	267 (81.4%)			1.40 (0.88–2.23)	0.148
More than 27	3 (1.7%)	6 (1.8%)			1.38 (0.32–5.86)	0.661
**Gender**						
Male	99 (57.5%)	172 (52.4%)	1.191	0.275	Reference	
Female	73 (42.4%)	156 (47.6%)			1.23 (0.84–1.78)	0.275
**Marital Status**						
Singe	98 (57%)	248 (75.6%)	22.364	**<0.001**	Reference	
Married	65 (37.8%)	67 (20.4)			0.41 (0.26–0.61)	**<0.001**
Divorced	7 (4.1%)	13 (4%)			0.73 (0.28–1.89)	0.522
Separated	2 (1.2%)	0 (0.0%)			0.00	
**University Type**						
Public	57 (33.1%)	112 (34.1%)	0.051	0.821	1.04 (0.71–1.54)	0.821
Private	115 (66.9%)	216 (65.9%)			Reference	
**Department/ Faculty**						
Science/ Biological Science	44 (25.6%)	164 (50%)	27.906	**<0.001**	2.74 (1.70–4.43)	**<0.001**
Business	72 (41.9%)	88 (26.8%)			0.90 (0.56–1.43)	0.659
Social Science	56 (32.6%)	76 (23.2%)			Reference	
**Living Arrangement**						
University hostel	42 (24.4%)	112 (34.1%)	5.062	0.080	1.56 (0.98–2.48)	0.056
Rented Home	60 (34.9%)	97 (29.6%)			0.95 (0.61–1.47)	0.822
With Family	70 (40.7%)	119 (36.3%)			Reference	
**Monthly Family Income**						
15 – 30k BDT	37 (21.5%)	140 (42.7%)	33.827	**<0.001**	4.64 (2.71–7.95)	**<0.001**
30 – 50k BDT	26 (15.1%)	54 (16.5%)			2.54 (1.37–4.71)	**0.003**
50 – 70k BDT	55 (32%)	90 (27.4%)			2.01 (1.19–3.38)	**0.009**
>70k BDT	54 (31.4%)	44 (13.4%)			Reference	
**Exercise**						
Never	59 (34.3%)	96 (29.3%)	1.611	0.447	1.23 (0.82–1.84)	0.303
Sometimes	102 (59.3%)	205 (62.5%)			1.51 (0.69–3.26)	0.297
Regularly	11 (6.4%)	27 (8.2%)			Reference	
**Sleeping Hours**						
<6 hours	72 (41.9%)	129 (39.3%)	0.301	0.583	Reference	
6–9 hours	100 (58.1%)	199 (60.7%)			1.11 (0.76–1.61)	0.583
**BMI**						
Underweight	16 (9.3%)	38 (11.6%)	0.930	0.818	1.53 (0.59–4.00)	0.379
Optimal	91 (52.9%)	175 (53.4%)			1.24 (0.56–2.76)	0.592
Overweight	54 (31.4%)	98 (29.9%)			1.17 (0.51–2.68)	0.704
Obese	11 (6.4%)	17 (5.2%)			Reference	

*Note: χ² = Chi-square test; OR = Odds Ratio; CI = Confidence Interval; FoMO = Fear of Missing Out; BMI = Body Mass Index; BDT = Bangladeshi Taka. Reference categories were used for logistic regression analysis. P-values < 0.05 were considered statistically significant.*

### Association Between Fear of Missing Out (FoMO) Levels with Behavioural, Psychological, and Smartphone Use Characteristics Among Participants

Out of the total respondents, individuals with high FoMo were significantly more likely to report poor sleep habits, late-night smartphone use, higher levels of depression, anxiety, stress, and smartphone addiction compared to those with low FoMo. Specifically, the proportion of participants who reported that using a smartphone kept them awake was higher among the high FoMo group (69.5%) than among the low FoMo group (77.9%), and this difference was statistically significant (χ² = 3.979, p = 0.046). Those who stayed awake after midnight were more likely to experience high FoMo (66.5%) than low FoMo (47.7%), showing a strong association (χ² = 16.597, p < 0.001). Participants who stayed awake past midnight were 2.17 times more likely to have high FoMo (OR = 2.17, 95% CI: 1.49–3.17, p < 0.001). No significant association was found between FoMo and smartphone use while walking (χ² = 0.385, p = 0.535), riding (χ² = 0.709, p = 0.400), or eating (χ² = 0.055, p = 0.814). In contrast, psychological variables demonstrated a very strong association with FoMo. Respondents with symptoms of depression were 7 times more likely to experience high FoMo compared to those without depression (OR = 7.01, 95% CI: 4.27–11.47, p < 0.001). Similarly, participants with anxiety symptoms were 5.61 times more likely to report high FoMo (OR = 5.61, 95% CI: 3.36–9.35, p < 0.001). The association between stress and FoMo was the strongest, where stressed individuals were 9.69 times more likely to have high FoMo (OR = 9.69, 95% CI: 6.33–14.84, p < 0.001). Furthermore, a significant relationship was observed between smartphone addiction and FoMo. Participants categorized as smartphone addicted were 4.46 times more likely to have high FoMo compared to those not addicted (OR = 4.46, 95% CI: 2.45–8.11, p < 0.001) (Table 5).

**Table 5:** Association between fear of missing out (FoMO) levels with behavioral, psychological, and smartphone use characteristics among participants.

Variable	Low FoMo	High FoMo	Χ^2^	P value	OR (95% CI)	P value
**Smartphone keep you awake**						
Yes	134 (77.9%)	228 (69.5%)	3.979	**0.046**	Reference	
No	38 (22.1%)	100 (30.5%)			1.54 (1.01–2.37)	**0.047**
**Awaking time**						
Up to 12 AM	90 (52.3%)	110 (33.5%)	16.597	**<0.001**	Reference	
After 12 Am	82 (47.7%)	218 (66.5%)			2.17 (1.49–3.17)	**<0.001**
**Using smartphone during walking**						
Yes	118 (68.6%)	216 (65.9%)	0.385	0.535	Reference	
No	54 (31.4%)	112 (34.1%)			1.13 (0.76–1.68)	0.535
**Using smartphone during riding**						
Yes	112 (65.1%)	201 (61.3%)	0.709	0.400	Reference	
No	60 (34.9%)	127 (38.7%)			1.17 (0.80–1.73)	0.400
**Using smartphone while eating**						
Yes	130 (75.5%)	251 (76.5%)	0.055	0.814	1.05 (0.68–1.62)	0.814
No	42 (24.4%)	77 (23.5%)			Reference	
**Depression**						
No	68 (39.5%)	28 (8.5%)	69.887	**<0.001**	Reference	
Yes	104 (60.5%)	300 (91.5%)			7.01 (4.27–11.47)	**<0.001**
**Anxiety**						
No	56 (32.6%)	26 (7.9%)	49.929	**<0.001**	Reference	
Yes	116 (67.4%)	302 (92.1%)			5.61 (3.36–9.35)	**<0.001**
**Stress**						
No	124 (72.4%)	69 (21%)	124.102	**<0.001**	Reference	
Yes	48 (27.9%)	259 (79%)			9.69 (6.33–14.84)	**<0.001**
**Smartphone Addiction**						
Addicted	14 (8.1%)	93 (28.4%)	27.410	**<0.001**	4.46 (2.45–8.11)	**<0.001**
Not addicted	158 (91.9%)	235 (71.6%)			Reference	

*Note: χ² = Chi-square test; OR = Odds ratio; CI = Confidence interval; FoMO = Fear of Missing Out. A p-value < 0.05 was considered statistically significant.*

## Discussion

The present study aimed to investigate Fear of Missing Out (FoMO) and smartphone‐related behaviors among university students in Bangladesh, and to examine how FoMO correlates with demographic, behavioral, and psychological variables, including depression, anxiety, stress, and smartphone addiction. Overall, the findings are consistent with a growing body of literature in both global and Bangladeshi contexts. They highlight the psychological burden associated with FoMO, particularly among specific subpopulations, and suggest that lifestyle factors such as sleep patterns and late-night phone use may be linked to well-being.

The prevalence and spread of FoMO in this sample were intriguing: moderate FoMO in the majority, 56.8%, low FoMO in approximately one third, and high FoMO 8.8%. Combined with smartphone addiction among approximately 21.4% of respondents, and very high rates of depression, anxiety, and stress. These numbers are generally in line with the earlier Bangladeshi literature, which observed that 61.4% of young adults in Bangladesh might be deemed as smartphone addicted; anxiety and depression were strongly linked with smartphone addiction [16]. Apparently, problematic Smartphone and Social Media Use Among Bangladeshi College and University Students During COVID-19, poor sleep, anxiety, and depression were also found to be predictors of problematic use of social media and smartphones [17]. Marital status, field of study: science/biological sciences, and lower family income were found to be significant in demographic predictors of high FoMO, whereas age, gender, sleep duration, exercise frequency, BMI, and living arrangement were not found to be significant. The observation that single students tend to report higher FoMO is consistent with international research. Studies suggest that individuals with lower offline social connections may rely more on online connectivity, which has been associated with higher FoMO and psychological distress [11]. The close relationship with academic discipline students of science/biological sciences with significantly higher odds of high FoMO) is notable. A significant amount of prior research has examined health/science students who frequently report high academic pressure with high smartphone addiction rates [18,19], but few studies have specifically explored FoMO by academic discipline. In contrast, smartphone use patterns and digital behaviors were related to smartphone addiction but did not further divide FoMO by field [16]. There is a close correlation between high FoMO and depression, anxiety, stress, and even smartphone addiction. Participants with high FoMO were approximately 7 times, 5.6 times, 9.7 times, and 4.5 times more likely to experience depression, anxiety, stress, and smartphone addiction, respectively, than those with low FoMO. This finding is aligned with previous research, where the relationship between FoMO and mobile phone addiction was found to be highly correlated [20]. Moreover, a significant correlation between problematic smartphone use and depression/anxiety after adjusting for age, sleep quality, etc [17]. Age, gender, sleep duration, exercise frequency, BMI, and living arrangement were not significantly correlated with FoMO level in this study. In contrast, studies from Switzerland and the Middle East found that smartphone addiction is significantly more prevalent among younger adolescents and young adults compared to older adults, with younger age being a strong predictor of problematic use [21,22]. Some research found that adolescents and university students report higher levels of FoMo, which is closely linked to their mobile and social media habits. High FoMO is significantly associated with poorer sleep quality, and both social media addiction (SMA) and social media fatigue (SMF) exacerbate the effect. The present results similarly show that sleep disturbance and late‐night phone usage after midnight are significantly associated with high FoMO. This parallel lends credibility to the notion that sleep hygiene is a crucial mediator in the FoMO‐psychological distress relationship. Also, the physical health findings, including eye pain, neck pain, shoulder pain, etc., also align with a study where smartphone addicted participants reported physical discomfort, eye strain, neck/shoulder pain [16]. Thus, existing literature indicates that excessive smartphone usage, especially at night and high-frequency usage, is linked to negative psychological and physical health results.

Considering the identified associations, some implications can be made. To start with, interventions at the university level could be helpful, such as the creation of awareness in relation to FoMO, tactics to decrease compulsive phone checking, especially in the evening, and the encouragement of healthy sleep habits. As an illustration, awareness campaigns may be used to raise possible issues associated with the overuse of smartphones, which include eye strain, musculoskeletal pain, and anxiety and depression symptoms. Second, FoMO and smartphone addiction may be included in mental health screening, psychological support, and mental health workshops, especially in science/biological students and those from lower socioeconomic backgrounds. Third, since no statistically significant results were found between variables like gender, age, and BMI and weight gain, the interventions may be oriented to be broad and inclusive and not be restricted to particular demographic groups. From a policy perspective, universities may consider adopting digital wellness strategies. These may include encouraging phone-free periods, particularly at night, promoting physical activity within academic programs, and facilitating offline social engagement through clubs and face-to-face activities. In addition, institutions may provide guidance on time management and the use of smartphone features such as Do Not Disturb mode or screen-time monitoring tools. Further, implications of the research, future investigations should be longitudinal research designs to unpack whether high FoMO causes depression/anxiety, or whether anxiety/depression causes FoMO bidirectional interaction. Similarly, qualitative work would be useful to investigate cultural, familial, and societal expectations on FoMO perceptions. The problem of measurement should also be mentioned to make cross-study results more legitimate; the definition of what is meant by high FoMO, smartphone addiction, thresholds, etc., needs to be standardized. This study establishes that FoMO is highly related to smartphone addiction, sleep disturbance, depression, anxiety, and stress among Bangladeshi university students. It recognizes single status, science/biological discipline, and low family income as especially significant demographic correlates. Sleep hygiene, mental health of university students, and decreasing compulsive smartphone behaviours appear to be the areas of intervention. Long-term follow-ups and cross-cultural research might focus our perception of directionality, modulators, and effective prevention interventions.

This study has some limitations, too. Causal inference cannot be made based on the cross-sectional design. Self-report measures can give rise to either recall bias or social desirability bias (students could underreport the harmful behaviors or symptoms). The sample, although quite large (n = 500), is only of university students and might not be representative of non-student young adulthood and older groups. Besides, Selection bias cannot be completely ruled out, even though the multistage stratified random sampling method was employed. The participation was voluntary and could have resulted in overrepresentation of some groups, especially those aged between 23 and 27 years, as indicated in the sample distribution. This may limit the generalizability of the findings. Others (e.g., frequency of exercise, duration of sleep) were not significantly associated; perhaps the measurement instruments were not sufficiently precise (e.g., categories too broad), or there was limited variation in the sample. Lastly, cultural and context considerations (e.g., family demands, Internet infrastructure, academic demands) may also mediate effects but were not significantly modelled in this research.

## Conclusions

The findings of this study indicate a distinct connection between excessive smartphone use and adverse consequences on their sleep quality and mental health in students. Results indicate the use of phones at late hours interferes with sleep cycles and is associated with stress, anxiety, and emotional burnout. Smartphone addiction has emerged as part of behavioural habits, but now it is an increasing health concern in the population that influences academic achievement and human life in general. This study highlights that raising awareness and behavioural change interventions may be helpful for university students to adopt healthy digital habits. Promoting minimal screen time, digital detox efforts, and effective stress management strategies may go a long way in enhancing the sleep and psychological well-being of students. Simply, smartphone responsibility and healthy digital engagement are key to improved mental health and academic performance in the digital age.

## Abbreviations

FoMo: Fear of Missing Out
MPA: Mobile phone addiction
PSU: Problematic Smartphone Use
HRV: Heart Rate Variability
SPA: Smartphone Addiction
SM: social media
PH: Psychological Health
DAS: Depression, Anxiety and stress
SES: Socioeconomic Status
CI: Confidence Interval
OR: Odds Ratio
SMF: Social Media Fatigue
BMI: Body Mass Index
CBT: Cognitive Behavioral Therapy
